# Effective plasmonic enhancement of up-conversion photoluminescence from $\alpha$-NaGdF_4_:Yb^3+^,Er^3+^ nanoparticles by gold dendrites

**DOI:** 10.1038/s41598-026-47244-9

**Published:** 2026-04-07

**Authors:** Ngoc Bao Tri Pham, Aliaksandr Burko, Valeryia Murashka, Diana Laputsko, Hanna Bandarenka, Alma Dauletbekova, Kahramon Mamatkulov, Grigory Arzumanyan

**Affiliations:** 1 Laboratory of Neutron Physics, Sector of Raman Spectroscopy, Joint Institute for Nuclear Research, Dubna, Russia; 2 Applied Plasmonics Laboratory, Belarusian State University of Informatics and Radioelectronics, Minsk, Belarus; 3 Department of Technical Physics, L.N. Gumilyov Eurasian National University, Astana, Kazakhstan; 4 University of Science, Viet Nam National University, Ho Chi Minh City, Viet Nam; 5 Institute for Lasers, Photonics, and Biophotonics, SUNY at Buffalo, Buffalo, NY USA

**Keywords:** Materials science, Nanoscience and technology, Optics and photonics, Physics

## Abstract

The current paradigm of plasmon-enhanced up-conversion photoluminescence (PE-UCPL) lays in intimate coupling domains of noble metal and lanthanide-doped nanoparticles. An increase of incident electromagnetic field and radiative emission rates is the key reason for the UCPL enhancement by the primitive plasmonic architectures of metal nanospheres/nanorods/nanoshells. Based on the hypothesis of precise tuning surface plasmon resonance in more complicated nanoobjects by adjusting process-structure relationship, we engineered a PE-UCPL platform composed of $\alpha$-NaGdF_4_:Yb^3+^,Er^3+^ nanoparticles and gold dendrites on macroporous silicon (macro-PSi). The uncertainty in contribution of metal dendrites to PE-UCPL is typically due to the structural unpredictability of their highly branched morphologies during formation. Remarkably, macro-PSi dramatically reduces such a barrier. An approach to manage dendritic morphology can be regarded as a controlled corrosive substitution of the silicon skeleton with gold atoms mediated by external fluorine ions from the HF-based electrolyte for gold deposition. Here, we present a comprehensive characterization of three types of Au dendrites grown on macro-PSi for selection of optimal geometry applied to enhance up-conversion of $\alpha$-NaGdF_4_:Yb^3+^,Er^3+^ nanoparticles. We particularly examined structural/optical properties of Au dendrites and explored a role of incident electric field projection using computer simulation of a dendrite composed of hexagonal bipyramids. The selected Au dendrites provided a broadband PE-UCPL upon 780–990 nm excitation accompanied by a 35-fold increase in a ‘red’ peak integrated intensity and a 26-fold enhancement in a ‘green’ one compared to reference $\alpha$-NaGdF_4_:Yb^3+^,Er^3+^ nanoparticles. Our results are prospective to advance many applications including bioimaging, solid-state lighting, and especially neutral-color solar cells based on macro-PSi.

## Introduction

Up-conversion photoluminescence (UCPL) is a multistep nonlinear optical phenomenon of absorbing low-energy photons—typically near-infrared (NIR)—and their conversion into higher-energy photons—visible or ultraviolet (UV)—which are then emitted. The art of managing the UCPL effect is essential to advance and achieve breakthroughs in many fields including photovoltaics^1–6^, low background biological imaging^7,8^, and phototherapy^9,10^ , to name a few.

Although up-conversion (UC) materials possess unique optical properties prospective for engineering radically new nanophotonic devices, they have one serious drawback. In more detail, the photoluminescence intensity of most UC composites along with lanthanide ions embedded in dielectric matrices—one of the most frequently studied up-converting systems—is rather low. It is even less prominent if nanostructured UC materials are used to shrink sizes of the end-system components. In this regard, a significant enhancement of the UC efficiency can be accomplished using plasmon resonances. That is why plasmon-enhanced up-conversion photoluminescence (PE-UCPL) has recently emerged as an important area of research bridging the fields of nanophotonics and materials science. This phenomenon involves exploiting surface plasmons—collective oscillations of free electrons in nanostructured metals. An enhancement of the photoluminescence efficiency through plasmon resonance is primarily attributable to the localized electromagnetic field effect facilitated by metallic nanostructures in close proximity to UC nanomaterials. This can lead to a substantial increase in both the incident electromagnetic field intensity and the radiative emission rates from UC nanomaterials^11,12^. In particular, the previously reported enhancement factors demonstrate the potential of plasmonic structures to dramatically elevate the photoluminescence output of the UC nanostructures^13^. Gold and silver are the most popular metals that have been considered for achieving the PE-UCPL effect^14^. It should be admitted that the combination of silver nanostructures with the UC nanomaterials is more beneficial because they exhibit stronger surface plasmon resonance (SPR) than gold ones, mainly due to their superior electromagnetic behavior in the visible region^15,16^. However, gold is selected as a material for PE-UCPL when modulation of plasmon resonance frequencies should be provided by adjusting the composition of plasmonic nanostructures. Namely, Ag nanoparticles (NPs) enable the SPR peak around $\tilde{4}00$ nm while in some cases optimal energy transfer processes between UC and plasmonic nanomaterials require more significant absorption in the spectral range over 500 nm typical for the SPR bands of Au NPs^17^. Additionally, gold is more preferable than silver since it exhibits chemical stability allowing long-term use in various applications without the degradation of plasmonic properties^11^. An engineering geometry of gold nanostructures has enabled tuning their SPR for the spectral overlap of excitation wavelengths of UC NPs, thus maximizing absorption and energy transfer efficiencies^18,19^. In particular, the electric field near gold nanostructures was found to increase the UC quantum yield by promoting energy transfer between the rare-earth elements (REE) composing the UC systems^20^. To date, a number of gold architectures that have shape-/size-tunable plasmon resonances such as nanoparticles, nanoshells, and nanorods have already opened new pathways to enhance UCPL of the REE-doped materials^15,21,22^. In the context of these results, it looks reasonable to exploit a PE-UCPL potential of more complex yet stronger plasmonic structures based on dendrites that are known to be the richest source of ’hot spots’—the sites of maximal electromagnetic field enhancement^23^. However, the effects of combining two or more intimately coupled domains of different UC NPs and plasmonic dendrites and their related applications remain underexplored. This is largely due to the difficulty of predicting a dendrite geometry during its growth and the character of the distribution and localization of the electromagnetic field in it upon infrared excitation.

Here, we make a contribution to fill this gap to a certain extent. Firstly, we fabricate gold dendrites (AuDNs) by corrosive deposition on a porous silicon template, which enables controllable metal nanostructuring as well as provides integration with the silicon substrate. Then, the different AuDN types are characterized for the selection of the dendritic morphology optimal to enhance UCPL of $\alpha$-NaGdF_4_:Yb^3+^,Er^3+^ NPs. The role of incident electric field projection on the plasmonic properties of AuDNs composed of hexagonal bipyramids forms is revealed by computer simulations. It is found that the selected AuDNs provide a broadband PE-UCPL upon 780–990 nm excitation accompanied by a 35-fold increase in the integrated intensity of the ‘red’ emission peak and a 26-fold enhancement in a ‘green’ one compared to the reference $\alpha$-NaGdF_4_:Yb^3+^,Er^3+^ NPs. The outlook of the engineered PE-UCPL platform can be fruitful for bioimaging, solid-state lighting, and solar cells with improved antireflective or neutral color features imparted by porous silicon^24,25^. In particular, these improvements directly address a key limitation in biomedical applications, where low UCPL intensity of bare UC NPs often restricts detection sensitivity. The amplified red/green emission signals under NIR excitation can facilitate deep-tissue bioimaging with minimal background interference^26^, while the compatibility of UC NPs with surface functionalization enables their use in targeted theranostics and NIR-triggered therapies^27^. The AuDNs/macro-PSi architecture thus represents a versatile strategy for amplifying UCPL performance across diverse technological domains.

## Results and discussion

### Morphology and plasmonic properties of Au dendrites on macroporous silicon

Electrochemical etching resulted in vertical cylindrical pore channels growing down to a depth of 5±0.5 μm in the silicon wafer. The pore diameter varied within the range of 0.5–1.7 μm, with a mean value of 1.2 μm. The number of pores was calculated to be 2,350 per square centimeter. According to the classification of porous media, such PSi is defined as a macroporous material^28^.

The mechanism of the electroless Au deposition on PSi is the reduction of metal ions due to electrons supplied by silicon atoms. It assumes the reduction of Au^3+^ cations to atomic Au^0^ by electron (e^–^) exchange with Si. The redox potential for Au^3+^/Au^0^ is positive, i.e. the Au deposition on the Si surface proceeds spontaneously as the result of the electron transfer from semiconductor valence band to the metal cations:

1 $${\mathrm{Au}}^{{3 + }} + 3{\mathrm{e}}^{ - } \to {\mathrm{Au}}^{0}$$

As a result of this process, Si is simultaneously oxidized since the above reduction reaction can also be viewed in terms of the holes (h^+^) “injection” into valence band of Si. In the presence of H_2_O, this injection induces the formation of silicon dioxide:

2 $${\mathrm{Si}} + {\mathrm{H}}^{2} {\mathrm{O}} + 4{\mathrm{h}}^{ + } \to {\mathrm{SiO}}_{2} + 4{\mathrm{H}}^{ + }$$

Consequently, long gold deposition is limited by the passivation of the silicon surface with silicon dioxide, which reduces the process rate. To overcome this hurdle, hydrofluoric acid (HF) can be added to the solution to dissolve the silicon dioxide, after which new charge carriers and water-soluble SiF_6_^2–^ ions are supplied:

3 $${\mathrm{SiO}}_{2} + 6{\mathrm{HF}} \to {\mathrm{SiF}}_{6}^{{2 - }} + 2{\mathrm{H}}^{ + } + 2{\mathrm{H}}_{2} {\mathrm{O}}$$

In these conditions, electroless deposition of Au on silicon is not limited by the growth of silicon dioxide (SiO_2_). New gold nuclei form on both the metal clusters that have already been adsorbed and the available silicon surface.

When considering a PSi surface instead of a monocrystalline one, the presence of Si-H_x_ groups plays an important role in Au deposition. These groups are usually generated via the hydrogenation of uncompensated Si bonds produced during electrochemical silicon etching. These hydrogenated bonds are highly reactive and can easily be oxidised, releasing additional electrons and thus accelerating Au deposition.

4 $$2{\mathrm{Si}} - {\mathrm{H}}_{{\mathrm{x}}} ({\mathrm{surface}}) + {\mathrm{H}}_{2} {\mathrm{O}} \to {\mathrm{SiO}}_{2} ({\mathrm{surface}}) + 2{\mathrm{H}}^{ + } + 4{\mathrm{e}}^{ - }$$

Therefore, PSi substrates can act as both a geometry-defining template for plasmonic nanostructures and a means of controlling redox reaction kinetics. This is an intriguing approach for producing metal nanoparticles and dendrites.

**Fig. 1:**
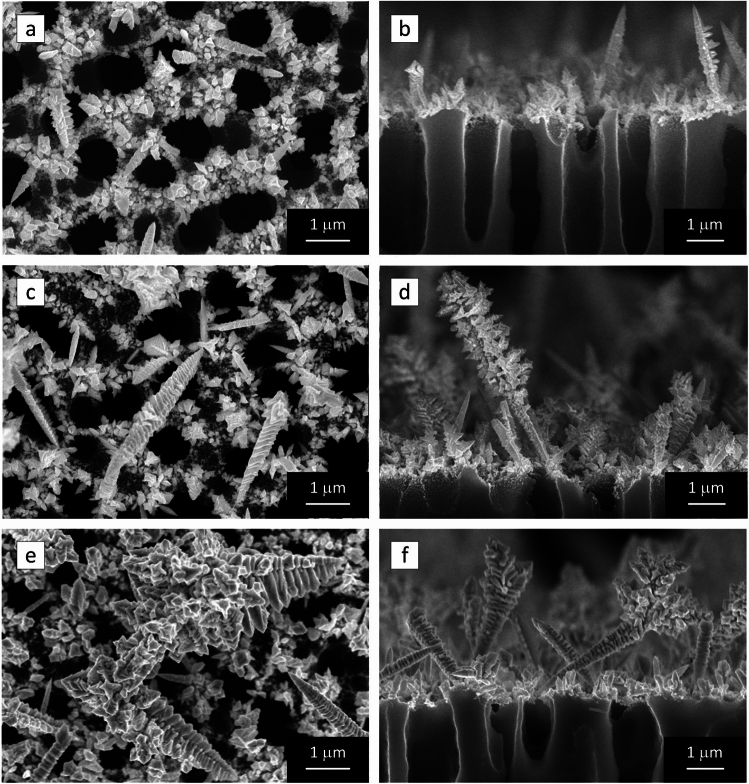
SEM-images of (**a**, **c**, **e**) top views and (**b**, **d**, **f**) cross sections of (**a**, **b**) AuDNs-01, (**c**, **d**) AuDNs-02, and (**e**, **f**) AuDNs-03 on macro-PSi.

Figure 1 shows scanning electron microscope (SEM) images of the surface and cross-section of AuDNs/macro-PSi samples formed at different concentrations of HF. The lowest concentration of HF results in the cover of the external surface of macro-PSi with Au particles and thorns (Fig. 1a, b).

**Fig. 2:**
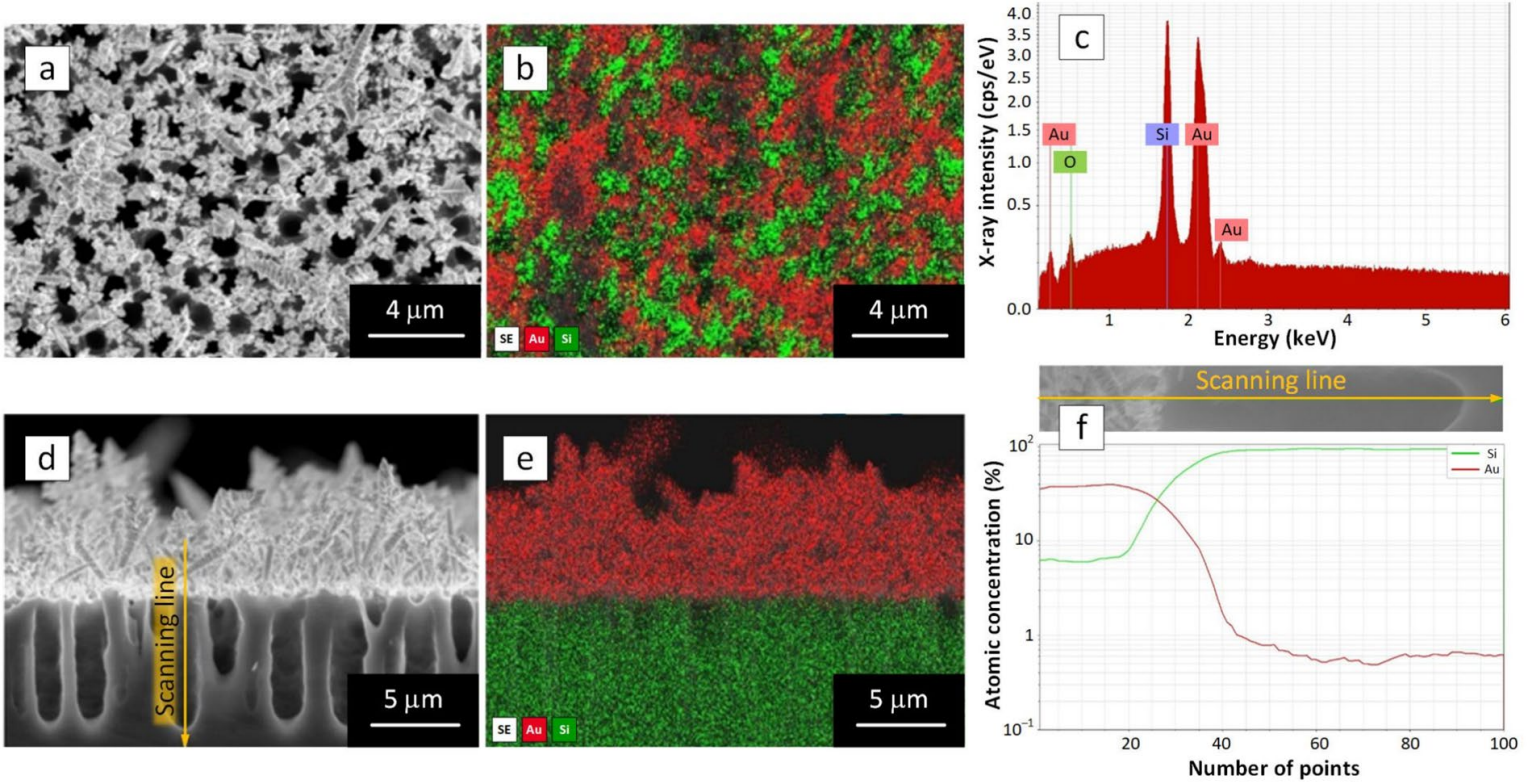
(**a**, **d**) SEM-images and (**b**, **e**) corresponding EDXS maps of the AuDNs/macro-PSi sample; (**c**) EDX spectrum was collected with the electron beam of approx. 1 μm diameter; (**f**) line scan shows no Au atoms in the pore.

The particles of 50–150 nm sizes densely coat the external surface of the macro-PSi skeleton. Some of the Au particles are partially coalesced in faceted agglomerates measuring 150–600 nm. At the same time, the thorns are rather short (maximal length does not exceed 1.5 μm) and rarer observed, which is definitely caused by the limited supply of the gold ions with electrons from silicon caused by the HF deficit. An increase of the HF concentration led to enrichment of the macro-PSi surface with the thorns that now resemble slightly branched dendrites (Fig. 1c, d). The highest volume of HF in the electrolyte enabled large Au dendrites to grow overlapping the pore mouths and Au particles (Fig. 1e, f). It is hypothesised that the Au structures start to grow according to the Volmer-Weber mechanism. The particle formation stage occurs due to the high density of the broken bonds on the macro-PSi surface. The initial Au particles act as seeds for the further growth of the separated Au thorns. The thorns then branch out, increasing in size and forming the dendrite-like structures. At this stage, the conditions required for the dendrite growth are met because the substrate contains the necessary precursors and simultaneously limits the delivery of the electrons from the Si atoms. The length of the dendrite trunks varies from 1.5 to 6.5 μm, whereas the dendrite branches are no longer than 100–700 nm depending on the HF concentration. It should be noted that the Au coatings contain nanoparticles and dendrite tips of a few nanometer sizes, which typically facilitate intense localized surface plasmon resonance (LSPR). The maximum LSPR can be expected at the tips of the thorns and dendrite branches. As can be seen in Fig. 2, which shows EDXS surface mapping and cross-section line scanning of the AuDNs-02 sample, it is evident that immersion of macro-PSi in the gold salt solution results in the formation of AuDNs exclusively on its outer surface. Energy dispersive X-ray spectroscopy (EDXS) analysis proves that the dendritic structures on the macro-PSi surface consist of only gold, free from any other element contaminations that could harm the plasmonic properties.

**Fig. 3:**
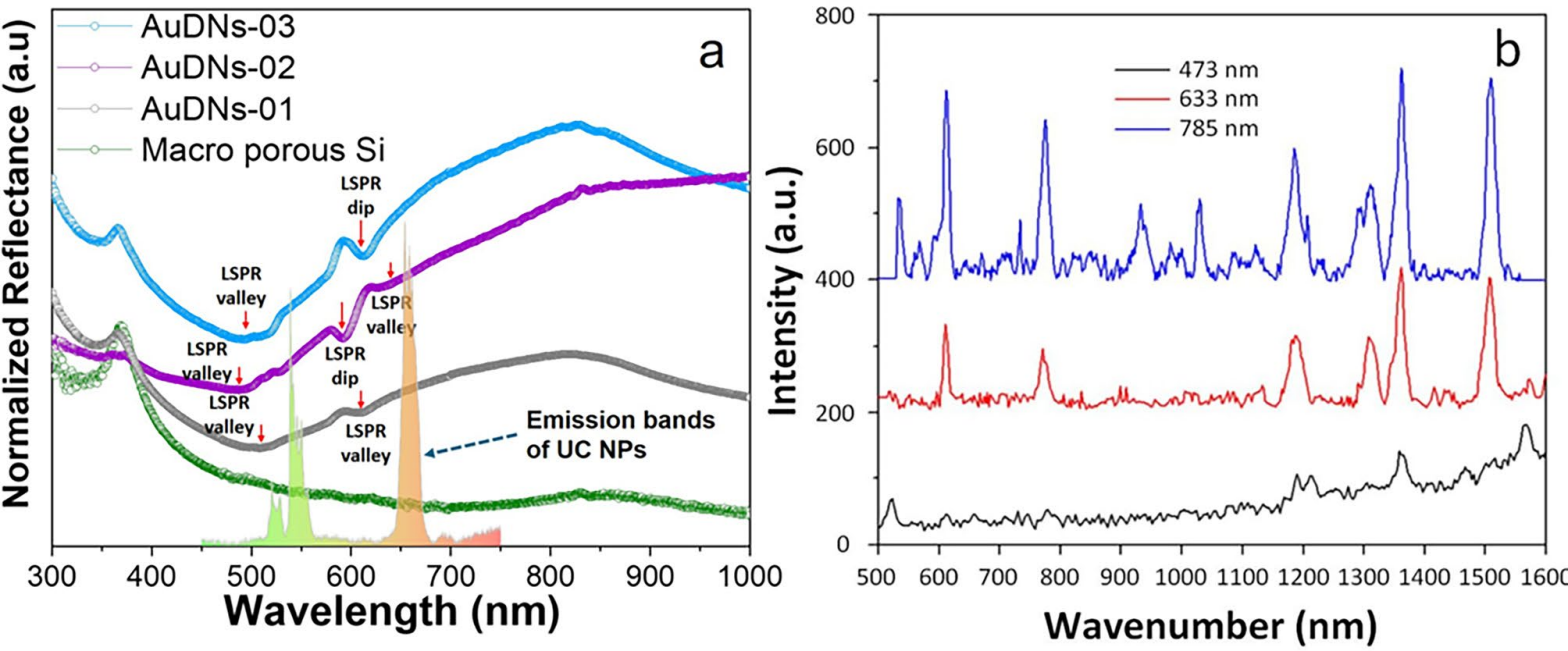
(**a**) Diffuse reflectance spectra of AuDNs-01, AuDNs-02, AuDNs-03 on macro-PSi and the bare macro-PSi reference, inset of the UCPL spectrum of UC NPs on AuDNs-02 under 800 nm excitation; (**b**) SERS spectra of 10^–6^ M R6G on AuDNs-02/macro-PSi.

Figure 3a shows the reflectance spectra of AuDNs/macro-PSi with varying structures. The AuDNs-02 structure (purple dots) exhibits one LSPR dip and two valleys: two broad valley features centered at approximately 400–460 nm and 630–810 nm, and an LSPR dip at 595 nm. Additionally, a slight reflectance enhancement is observed between 810 nm and 1000 nm, which is an atypical trend compared to AuDNs-01, AuDNs-03, and bare macro-PSi. This may be due to an extended plasmon tail that extends into the NIR region. In contrast, the AuDNs-03 sample (light blue dots) exhibits only an LSPR valley around 400–460 and an LSPR dip at 625 nm. The AuDNs-01 sample (black dots) exhibits a single shallow LSPR valley. These spectral features are assigned to interband transitions from the conduction band to the d-band. The red-shift observed in the second LSPR dip from the AuDNs-02 sample to the AuDNs-03 sample could be due to an increase in the size of the dendritic structure^29^. Consequently, the AuDNs-02 structure demonstrates enhanced spectral overlap capability for enhancing the LSPR excitation across the studied spectral range. Thereby, it was selected as the most promising plasmonic substrate for enhancing UCPL.

Additional tests were carried out using surface-enhanced Raman scattering (SERS) spectroscopy to double-check the plasmonic properties and the LSPR band of the AuDNs-02 sample. Figure 3b shows the SERS spectra of rhodamine 6G (R6G) molecules adsorbed onto the surface of AuDNs-02/macro-PSi. The maximal SERS-activity is clearly evident when the sample is excited by laser radiation with a wavelength of 785 nm. This is due to LSPR in the NIR range, as was discovered when studying the diffuse reflectance spectra.

The shift of the excitation radiation to the shorter wavelength results in the lower SERS-activity, and when a blue laser is used, the analyte spectrum is practically not identified.

Furthermore, the latter case is characterized by the combustion of organic molecules, as evidenced by an increase in background in the region of the 1366 and 1580 cm^−1^ bands responsible for the Raman scattering of amorphous carbon.

### Morphology of Au dendrites on macroporous silicon decorated with $\alpha$-NaGdF_4_:Er^3+^,Yb^3+^ nanoparticles

**Fig. 4:**
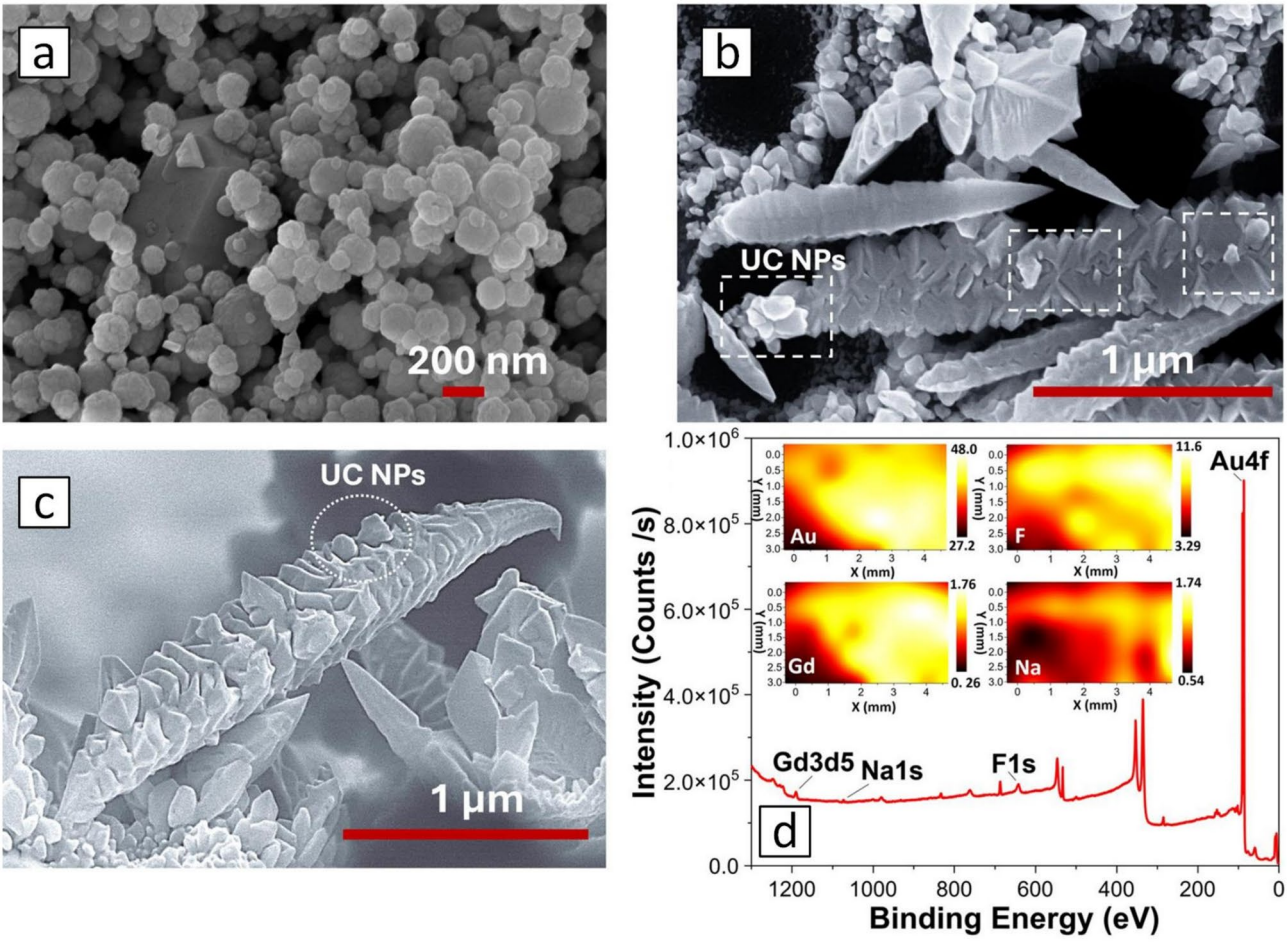
(**a**) The SEM-image of $\alpha$-NaGdF_4_: Yb^3+^, Er^3+^ NPs; (**b**–**c**) The SEM images of UC NPs on AuDNs-02/macro-PSi: top-view and cross-section view, inset highlights the region of UC NPs distribution; (**d**) XPS survey spectrum confirming the elemental composition of the UC NP-coated AuDNs-02/macro-PSi substrate, inset: spatially resolved XPS elemental maps.

As shown in the SEM image (Fig. 4a), the synthesized $\alpha$-NaGdF_4_:Er^3+^,Yb^3+^ NPs exhibited a quasi-spherical shape with an average diameter of 230 nm. Figure 4b, c highlights that the UC NPs are well-located on the branches of the AuDNs grown over the macro-PSi layer, whose pores appear as the black spots of 0.5–1.7 μm. Additionally, some UC NPs are located at the tips of the dendritic branches. This suggests strong coupling between the AuDNs and UC NPs, driven by the enhanced electromagnetic field localized at the tips and branches of AuDNs.

The X-ray photoelectron spectroscopy (XPS) results (Fig. 4d) clearly establish the elemental composition of AuDNs-02/macro-PSi that are coated with UC NPs. The core-level spectra exhibit distinct peaks corresponding to gold (Au 4f) from the AuDNs, and gadolinium (Gd 3d5), sodium (Na 1s) and fluorine (F 1s) from UC NPs. Spatially resolved elemental maps (Fig. 4d, inset) demonstrate a strong and uniform Au signal distributed over the entire substrate area. Importantly, the Gd, Na, and F signals, characteristic of the UC NPs, are also uniformly distributed and co-localized with the Au signal. This confirms that the UC NPs form a complete and homogeneous coating across the AuDNs substrate surface.

### Plasmon enhanced up-conversion photoluminescence spectra of Au dendrites

**Fig. 5:**
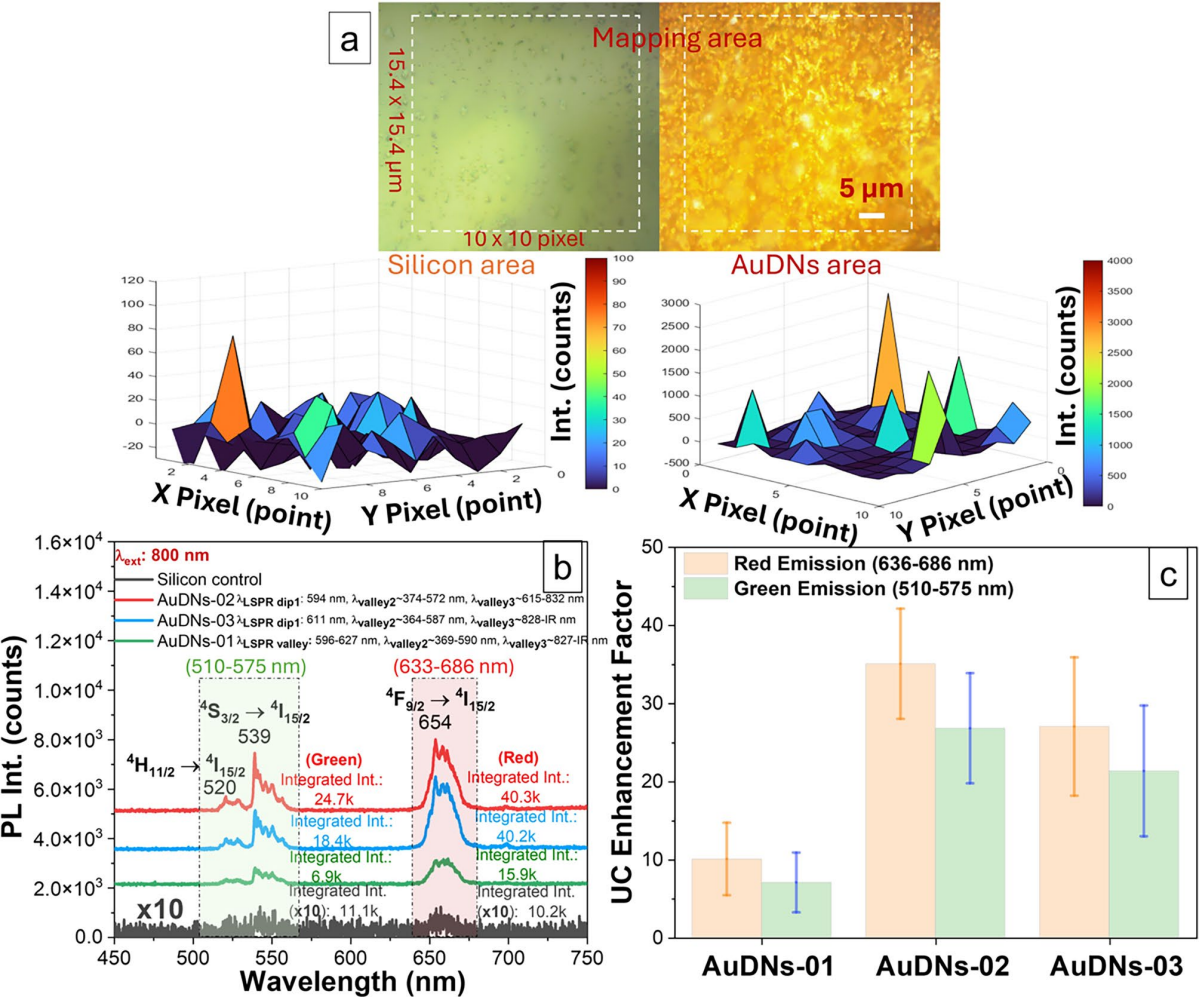
(**a**) Micrograph and PL mapping (integrated at 650–657 nm-red emission band) of UC NPs deposited on a silicon substrate and AuDNs-02 substrate under 800 nm excitation. (**b**) Up-conversion photoluminescence (UCPL) spectrum of the UC NPs coupled with gold dendritic nanostructures (AuDNs-01, AuDNs-02 and AuDNs-03) and a silicon reference, green emission band (integrated between 510–575 nm) and red emission band (integrated between 633–686 nm). (**c**) UCPL enhancement factors for AuDNs relative to the non-plasmonic control case (UC NPs on silicon substrate), error bars represent standard deviations derived from enhancement factor measurements with five randomly plotted positive pixels on a UCPL map of the plasmonic and non-plasmonic samples, respectively.

To verify the LSPR-mediated enhancement of UCPL in Au dendritic nanostructures, we measured the UCPL spectra of UC NPs coupled with AuDNs samples and a silicon reference substrate. Figure 5a shows a significant UCPL enhancement for UC NPs on AuDNs across multiple regions under 800 nm excitation. Specifically, UC NPs deposited on AuDNs exhibited UCPL intensities ranging from approximately 1000 to 3000 counts, compared to <100 counts for UC NPs on silicon. As shown in Fig. 5b, UC NPs exhibited significantly enhanced emission the green (510–550 nm) and red (640–680 nm) spectral regions across all plasmonic configurations compared to the non-plasmonic reference when excited at 800 nm. These emissions correspond to three transitions of Er^3+^ ions ^2^H_11/2_$\rightarrow$^4^I_15/2_ (green), ^4^S_3/2_$\rightarrow$^4^I_15/2_ (green), and ^4^F_9/2_$\rightarrow$^4^I_15/2_ (red)^30^. The low UCPL intensity from uncoupled UC NPs confirms their weak intrinsic activity at 800 nm excitation.

The maximum enhancement occurred in AuDNs-02 and AuDNs-03, where AuDNs-03 exhibited red emission dominating over green, and AuDNs-02 showed red emission comparable to green, both yielding Red/Green ratios >1. This preferential red enhancement originates from the modification of UCPL dynamics (e.g., altered decay rates via modulation of the local density of optical states^31,32^) induced by strong local fields of the plasmonic AuDNs structure. Such wavelength-selective enhancement is consistent with reports on other plasmonic nanostructures^33,34^. The observed preferential enhancement of the red emission over the green emission in AuDNs-02 can be understood by considering the interplay between the Er^+3^ energy level characteristics and the plasmonic electric field distribution. The green emission originates from the ^4^S_3/2_$\rightarrow$^4^I_15/2_ (green) transition, while the red emission arises from the ^4^F_9/2_$\rightarrow$^4^I_15/2_ transition (Fig. 9). These levels exhibit different radiative decay rates and non-radiative relaxation pathways. Time-resolved measurements in recent literature further validate this mechanism: plasmon-UC NPs coupling reduces both rise and decay times relative to uncoupled systems. Notably, the stronger reduction observed for red emission implies faster excited-state population and enhanced radiative decay rates, resulting in intensified UCPL with predominant red emission^34^. Furthermore, the plasmon resonance of the AuDNs creates a localized electric field that is wavelength-dependent. Based on the spectral overlap analysis (Fig. 3a and the inset of 3a), the enhanced red emission can be attributed to the favorable spectral overlap between the second LSPR valley of AuDNs-02 and the red emission band (660–680 nm), where the resonance dip is closely aligned. Conversely, although the first LSPR valley shows some overlap with the green emission (530–570 nm), the substantial spectral detuning between its resonance dip and the green band results in weaker plasmonic coupling for this transition. According to the Purcell effect, this stronger overlap increases the local density of optical states for the red transition more significantly, thereby enhancing the radiative decay rate of the red emission relative to the green. This wavelength-selective enhancement is consistent with previous reports on plasmon-coupled up-conversion systems^35,36^, where the spectral matching between plasmon resonance and emission bands plays a dominant role in determining the color tuning ratio. Statistical analysis across multiple sample positions (Fig. 5c) reveals that AuDNs-02 is the optimal structure for LSPR-mediated UCPL enhancement, achieving enhancement factors of up to 35-fold (red) and 26-fold (green). This suggests AuDNs-02 generates the strongest local fields under 800 nm excitation, resulting in the greatest LSPR-mediated UCPL enhancement. The UCPL enhancement factor is defined as the ratio of the integrated UCPL intensity from UC NPs on AuDNs plasmonic structures to that from UC NPs on silicon substrate (non-plasmonic case). Integration ranges are 510–575 nm for the green emission and 633–686 nm for the red emission bands of Er^3+^.

**Fig. 6:**
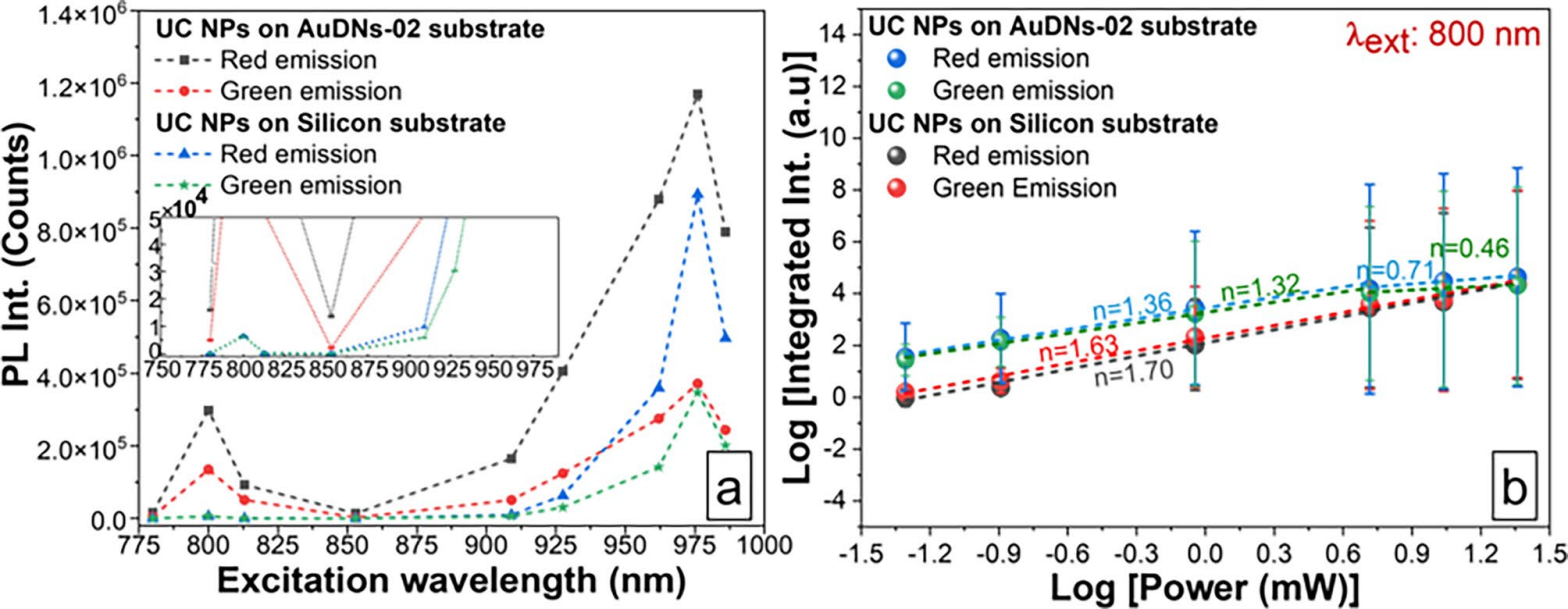
Photoluminescence properties of AuDNs-02 coupled with UC NPs: (**a**) UCPL excitation spectrum, inset: Magnified view of the low-intensity spectral regions. (**b**) Double-logarithmic plot of UCPL intensity versus excitation power, error bars represent standard deviations derived from integrated intensity measurements with three independent measurement spots.

Figure 6a shows a comparison of the photoluminescence intensity in dependence on the excitation wavelength for the UC NPs on AuDNs-02 and the UC NPs on Si. The graph for bare UC NPs exhibits a sharp peak at 976 nm (Yb^3+^ absorption), with a full width at half maximum (FWHM) of $\sim$10 nm. Upon coupling, the 976 nm peak intensifies by $\sim$1.8$\times$ for red emission but remains almost unchanged for green emission. Additionally, the overall band from 850–990 nm broadens to $\sim$15–20 nm, a shoulder emerges at 940–950 nm, and a smaller secondary peak appears near 800 nm. The enhancement of red emission consistently exceeds green enhancement across all wavelengths, indicating selective plasmon enhancement favoring the (^4^F_9/2_$\rightarrow$^4^I_15/2_) transition^34^. This is consistent with the reported preferential acceleration of the red-emitting-state population and the greater lifetime reduction in plasmon-coupled systems.

AuDNs provide broadband UCPL enhancement across the 780–986 nm wavelength range. At plasmon-excitation wavelengths (e.g., 780 nm, Fig. 3), where UC NPs exhibit no Er^3+^/Yb^3+^ absorption (with a silicon reference showing negligible UCPL intensity at 780/ 853 nm excitation), measurable amplification persists (see the inset in Fig. 6a). In contrast, at the maximum absorption wavelengths of UC NPs (910–990 nm), where AuDNs plasmon coupling is suboptimal, the UCPL intensity reached its peak value despite modest plasmonic enhancement.

At lower wavelength than 976 nm, UCPL process likely occurs through the direct excitation of Er^3+^^37,38^ and plasmon-activated pathways^33^, leveraging the overlap of the residual field with the absorption of Er^3+^ ions. Notably, UCPL of UC NPs seems to be negligible without the assistance of plasmons, thus indicating the important role of plasmon-activated pathways.

Figure 6b illustrates the power-dependent UCPL for different samples. The UCPL intensity (I_UCPL_) follows a power law

5 $${\mathrm{I}}_{{{\mathrm{UCPL}}}} \propto {\mathrm{P}}^{{\mathrm{n}}} ,$$

where *n* represents the number of pump photons absorbed per emitted visible photon. Another term is the number of photons involved in the UCPL process^22^. For conventional UC NPs on silicon, both green (^4^F_9/2_$\rightarrow$^4^I_15/2_) and red (^4^S_3/2_/^2^H_11/2_$\rightarrow$^4^I_15/2_) emissions exhibit a quadratic power dependence with *n* $\approx$ 2, confirming a two-photon process in Er^3+^.

In plasmonic structures, however, *n* decreases significantly under weak excitation (Fig. 6b), deviating from *n =* 2. This reduction stems from surface plasmon-induced local field enhancement, which saturates multiple excited states of Er^3+^ and alters UCPL kinetics^32^. Saturation originates from competition between the linear decay and upconversion pathways in these states^22^. At high excitation power,* n* $\approx$ 1 for both emission bands, indicating a transition to single-photon-dominated regime. This shift is attributed to complete saturation of the ^4^I_11/2_ and ^4^F_7/2_ levels^39^, driven by plasmon-mediated amplification of the local electromagnetic field^22,40^. Notably, plasmonic nano-antennas induce local heating through photothermal conversion, exacerbating thermal quenching of UCPL in NaYF_4_:Yb^3+^, Er^3+^ NPs^22,34^. However, for the specific $\alpha$-NaGdF_4_:Yb^+3^,Er^+3^ UC NPs employed herein, recent temperature-dependent UCPL study^41^ indicate that luminescence intensity changes are negligible within the 50–100 °C range, which corresponds to the expected temperature range of plasmon-induced local heating. Notably, a significant enhancement of the thermal emission signal was also observed at temperatures above 227 °C. Consequently, thermal quenching attributable to local heating from AuDNs is unlikely to substantially contribute to the power-dependent behavior observed here. This supports our interpretation that excited-state saturation constitutes the primary mechanism governing the observed decline in the photon number *n*.

### The electric field of the Au dendrite structure and the mechanism of plasmon-enhanced up-conversion photoluminescence

**Fig. 7:**
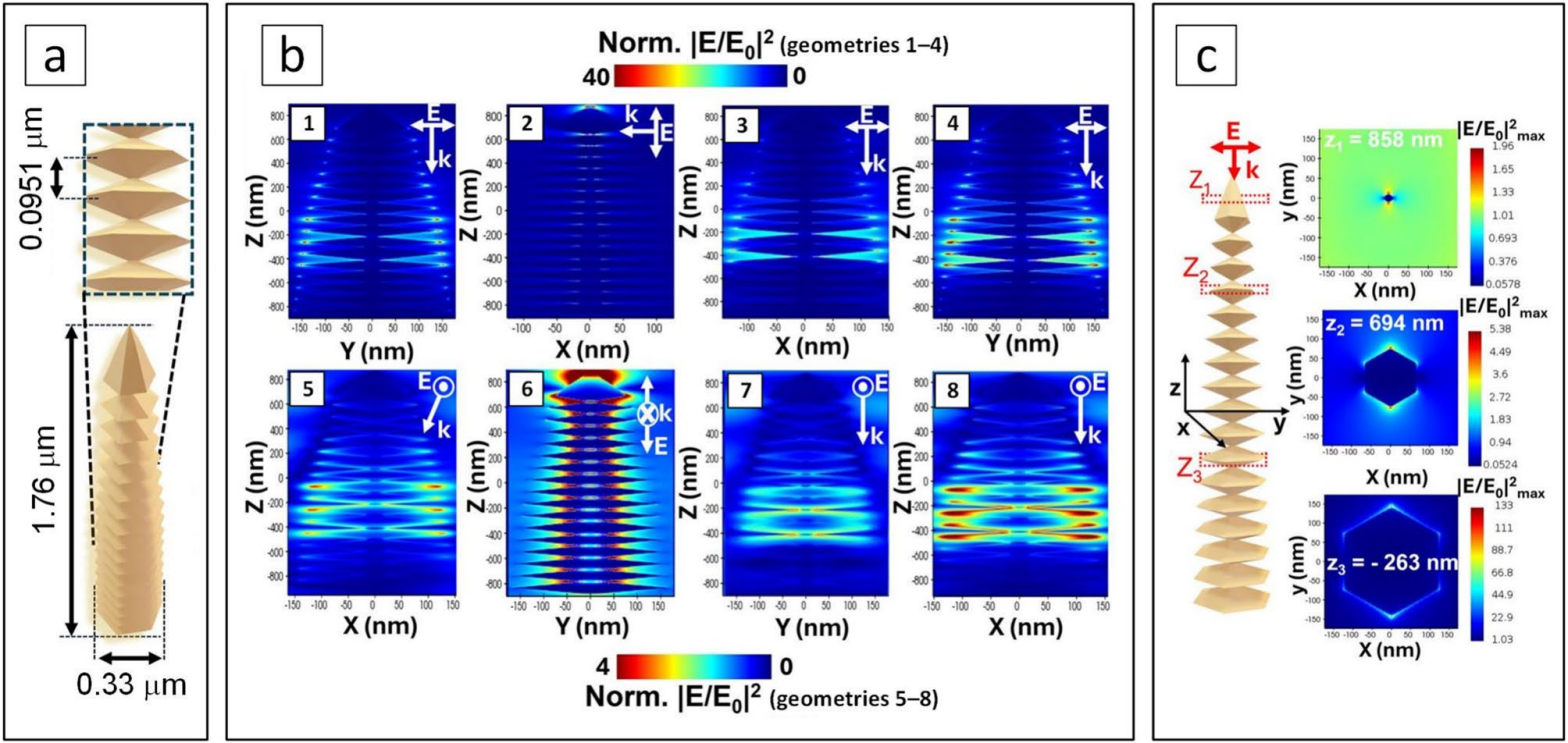
Simulation of the single AuDN-02 geometry and the electric field in the AuDN model: (**a**) 3D structural model of AuDN-02; (**b**) electric field distribution in AuDN-02 under 800 nm excitation wavelength for various propagation geometries including top-down projection at the angle of 30 degrees to z axis (1, 5), orthogonal projection to the dendritic plane (2, 6), top-down projection with x-polarization (3, 7) and y-polarization (4, 8); (**c**) 3D structural model of AuDN-02 with selected xy cross-sections along the dendrite trunk.

The 3D model of AuDNs-02 was constructed to reflect its realistic morphology (Fig. 1c, d) incorporating the parameters determined from the ImageJ analysis. With average length about 1.76±0.60 μm and the length of branch about 0.33±0.13 μm, the spacing between branches was 0.0951±0.0278 μm (Fig. 7a).

The morphological diversity of AuDNs-02/macro-PSi which includes vertical (z-axis-aligned), horizontal (xy-plane-parallel), and inclined dendritic orientations, yields varied electric field responses under fixed-wavelength laser excitation (Fig. 7a, b). Anisotropic electric field distributions arise from structural asymmetries between the zx- and zy-cross-sectional planes. Consequently, systematic cross-sectional analysis of 1–8 geometries (Fig. 7b) reveals the mechanisms of electric field localization and enhancement across the selected AuDN-02 structural model. Notably, geometries 2, 6 and 4, 8 (Fig. 7b) exhibit intense LSPR modes, generating >40-fold electric field enhancement predominantly at dendritic tips and proximal to symmetry axes due to the lightning rod effect.

The periodic structural features in geometry 4, 8 (Fig. 7b) further demonstrate multidimensional electric field propagation. Cross-sectional profiling in the xy plane (Fig. 7c) reveals stronger electric field localization on the larger dendritic branches compared to their smaller counterparts. The maximum electric field located on the larger branches can be 25 times stronger than on the smaller ones.

**Fig. 8:**
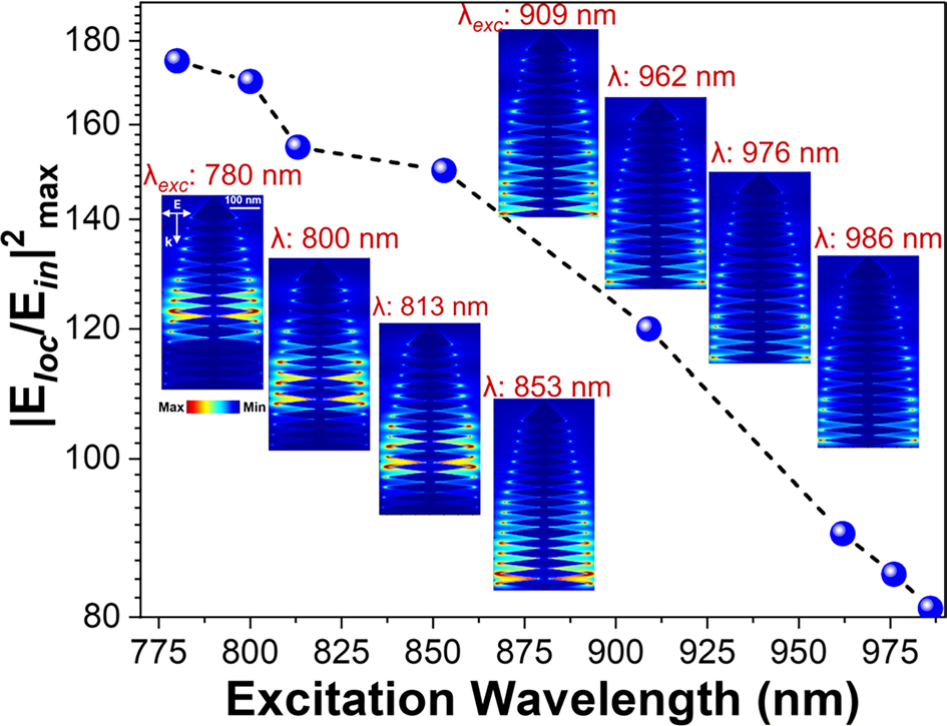
Wavelength-dependent electric field enhancement factor of AuDNs-02 (logarithmic scale). Inset: Spatial distribution of the electic field cross-section in a top-down projection under y-polarized illumination.

The electric field maximum enhancement factor on a logarithmic scale exhibits a decreasing trend with increasing excitation wavelength. The intensity at 986 nm in the NIR region remains significant, still reaching nearly half the peak value measured at 780 nm (Fig. 8). The strongest electric field enhancement region occurs between 780 and 850 nm, which corresponds to the LSPR of AuDN (Fig. 3). Within the NIR region, electric field enhancement persists, decreasing almost linearly with red-shifted wavelengths. This is attributed to the inter-band absorption tail extending into the NIR, which absorbs excitation energy and generates localized field amplification around the AuDN surface, albeit weaker than the primary LSPR modes. Spatial electric field distributions under various excitation wavelengths illustrate the evolution of the AuDN response as the wavelength shifts from the visible to the NIR region (Fig. 8). The highly localized fields between AuDN branches shift from the center of the structure towards its ends. This shift occurs because longer wavelengths preferentially excite larger branches rather than smaller ones. As excitation moves further into the NIR, the electric field amplitude diminishes and becomes strongly localized only at the branch tips.

In the PE-UCPL mechanism, the interactions between the rare-earth luminescent centers and the surface plasmons are critical. Plasmonic nanostructures such as AuDNs generate intense electric fields that deeply influence neighboring UC NPs. When positioned at plasmonic ’hot spots’, these electric fields modify the local density of optical states. This alters the electronic transitions within the luminescent centers, thereby enhancing either the absorption cross-section or the radiative decay rate in optimally coupled systems^11^. The intensity of UCPL exhibits a fourth-power dependence on the intensity of the near-field electric field. Consequently, plasmonic field enhancement plays a key role in governing PE-UCPL processes by modulating: radiative and non-radiative decay rates, absorption cross-sections and energy transfer coefficients. For monochromatic excitation, the absorption cross-section scales with $\left| {{\mathrm{E/E}}_{0} } \right|^{2}$. Optimal PE-UCPL efficiency requires spectral alignment between: (1) the plasmon resonance in the metallic nanostructure and the emission bands of the rare-earth ions, (2) the excitation wavelength and plasmonic absorption/emission resonances^31^.

**Fig. 9:**
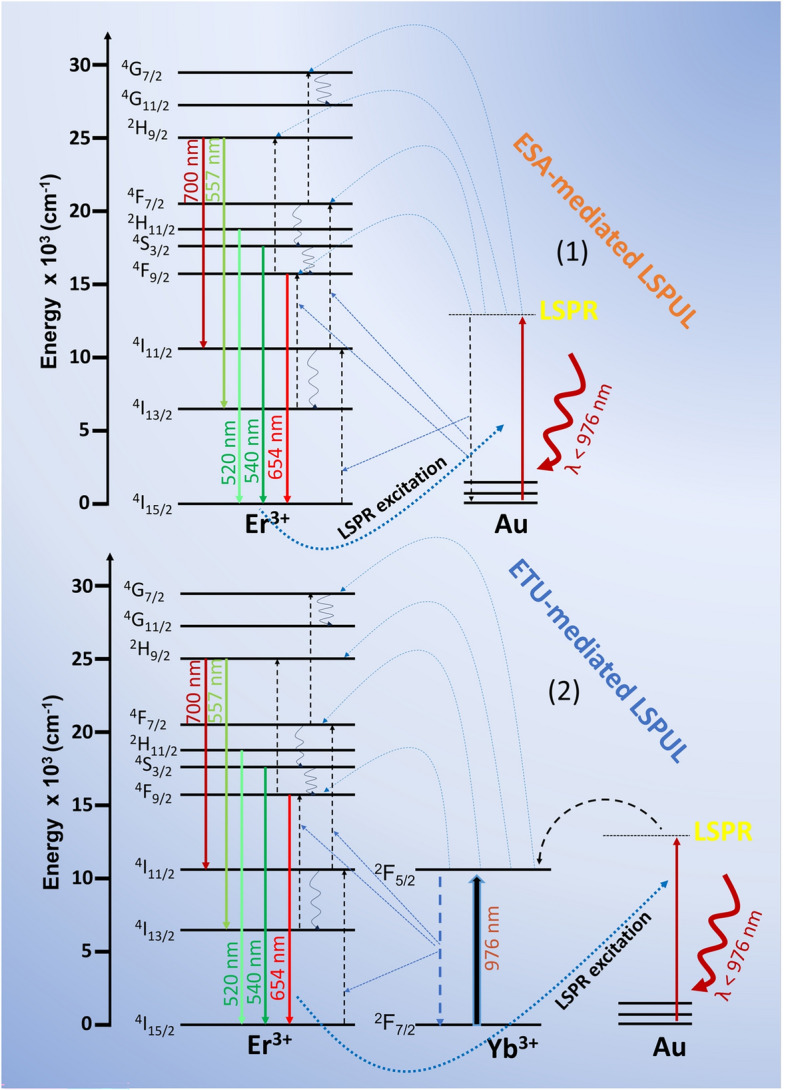
Schematic diagram of the two-paths interaction between the LSPR effect of AuDNs and the energy-transfer UC process of $\alpha$-NaGdF_4_:Yb^3+^,Er^3+^ NPs.

This study satisfied two critical conditions for PE-UCPL strategically. Firstly, AuDNs were fabricated and selected to align their LSPR absorption dip spectrally (Fig. 6) with the green/red emission bands of UC NPs maximizing near-field enhancement and PE-UCPL efficiency (Fig. 9).

Secondly, excitation at 800 nm simultaneously satisfied the following two conditions: (i) plasmon resonance conditions in the AuDNs and (ii) UCPL excitation requirements for the UC NPs. This dual-resonance approach enables efficient energy utilization by coupling plasmonic field enhancement with direct UC activation.

The electric field distribution of AuDNs exhibits strong enhancement under 780–860 nm excitation but remains inactive at longer NIR wavelengths (Fig. 9). This explains the high PE-UCPL efficiency observed at 800 nm (Fig. 6a), which confirms the dual-resonance strategy. While 976 nm excitation optimally activates Yb^3+^ (^2^F_7/2_ $\longrightarrow$ ^2^F_5/2_ transition) in UC NPs on silicon (maximizing red/green emission; blue/green dashed lines, Fig. 6a), this wavelength does not match the plasmon resonance of the AuDNs. Consequently, the PE-UCPL efficiency at 976 nm remains lower than at 800 nm.

Although Yb^3+^ cannot absorb photons below 976 nm (Fig. 9), weak luminescence persists in UC NPs/silicon under excitation at 780, 800, 813, 853, 909, and 956 nm. The same senario was also observed in previous reports for $\alpha$-NaGdF_4_:Yb^3+^,Er^3+^ system under 800 nm excitation^42^. The most effective approach to activating the UCPL of UC NPs under excitation lower than 976 nm is Nd^3+^ co-doping, where Nd^3+^ acts as a sensitizer. Its excited state which is higher than the ^2^F_5/2_ level of Yb^3+^, enables the efficient absorption of higher-energy photons. Notably, plasmonic nanostructures can amplify this weak emission without the need for Nd^3+^ sensitizers (Fig. 6a), which demonstrates their unique ability to enhance non-resonant UCPL processes.

These observations support the localized surface plasmon-enhanced upconversion luminescence (LSPUL) mechanism^33^, which occurs via two distinct pathways:

1. Excited-State Absorption (ESA)-mediated LSPUL: In Er^3+^-doped systems, the ladder-like energy levels enable sequential plasmon absorption. Energy matching occurs between plasmons and the Er^3+^ transitions (^4^I_15/2_-^4^I_11/2_, ^4^I_13/2_-^4^F_9/2_, ^4^I_11/2_-^4^F_7/2_, ^4^F_9/2_-^2^H_9/2_ and ^4^F_7/2_-^4^G_7/2_) promoting electrons to higher states (Fig. 9, path 1). Plasmon sensitization alone (without Yb^3+^) allows sequential absorption from the ^4^I_11/2_ to the ^4^F_7/2_, followed by emission at 520/540 nm via multiphonon relaxation. Emissions requiring nonradiative relaxation from ^4^I_11/2_^4^I_13/2_ (410, 557, 700 nm) are suppressed, while the 654 nm emission remains prominent due to an alternative relaxation channel from^4^F_7/2_^4^F_9/2_.

2. Energy Transfer Up-conversion (ETU)-mediated LSPUL: In Yb^3+^/Er^3+^ co-doped systems, plasmons resonantly excite Yb^3+^ ions. Yb^3+^acts as a sensitizer, transferring energy to Er^3+^ via Förster resonance energy transfer (Fig. 9, path 2). This pathway is dominant due to the efficient coupling of plasmon-Yb^3+^ ions coupling and the longer excited-state lifetime of the Yb^3+^ ion. The plasmon energy populates Yb^3+^ (^2^F_5/2_) state, which then excites Er^3+^ ion to ^4^I_11/2_, ^4^F_7/2_ or ^4^G_7/2_ states. This alleviates the ^4^I_11/2_^4^F_13/2_ relaxation bottleneck, thereby enhancing emissions at 410, 557, 655, and 700 nm. Additionally, emissions at 470/510 nm (from ^4^G_7/2_ and ^4^G_11/2_^4^I_13/2_) intensify due to the increased density of high-energy photon states under ETU-LSPUL.

The achieved UC enhancement factors of 26-fold (green) and 35-fold (red) are substantial (Fig. 5c), surpassing or comparative the performance of numerous plasmonic structures reported in the literature (Table 1). This enhancement is attributed to the unique plasmonic properties of AuDNs on macroporous Si, which were fabricated via a simplified and cost-effective method. This approach facilitates intense near-field plasmonic coupling, thereby enabling remarkable enhancement of upconversion luminescence.

Although the current steady-state spectroscopic data strongly support an excitation-enhancement mechanism, we acknowledge that directly probing modifications to emission dynamics would require time-resolved photoluminescence (TRPL) measurements. Systematic TRPL studies thus represent an important direction for future work aimed at decoupling excitation and emission contributions to fully elucidate the dynamic interplay between plasmonic near-fields and lanthanide emission dynamics.

Although direct TRPL measurements were not performed in this study, mechanistic insights can be drawn from analogous plasmon-UCNP systems reported in the literature^34^. For instance, recent work has observed reduced rise and decay times ($\tau$_rise_, $\tau$_decay_) in UCPL when UC NPs were coupled with gold nanostructure arrays compared to isolated UC NPs, a trend widely corroborated in gold plasmonic structures^43^. Physically, the shortened rise time signifies faster population of the excited energy levels, consistent with enhanced excitation rates driven by plasmonic near-field amplification. Conversely, the reduced decay lifetime is commonly associated with the introduction of additional decay channels by the gold metallic environment, which modifies both radiative and non-radiative transition rates^44,45^. Crucially, when such lifetime reduction correlates with enhanced UCPL intensity–as documented in these studies and mirrored by the substantial enhancement factors observed in our AuDNs/macro-PSi system–it suggests that the plasmonic coupling primarily boosts the radiative decay rate rather than introducing dominant non-radiative losses. This literature-supported inference aligns with our observation of significant UCPL intensity enhancement, implying that emission dynamics are favorably modified alongside excitation enhancement.

The plasmon-enhanced UCPL strategy demonstrated here is expected to be generalizable to other high-efficiency hosts, including $\beta$-NaYF_4_. Since the core mechanism–local electromagnetic field amplification–depends primarily on spectral overlap rather than host-specific crystallography, the design principles established herein could be readily transferable. This view is supported by prior reports of effective plasmonic coupling in $\beta$-NaYF_4_-based systems^33,46^, suggesting broad applicability across diverse UC NPs platforms.

**Table 1:** Comparison table of PE-UCPL performance.

Plasmonic structure	Preparation method	UC enhancement factor	Excitation wavelength	References
Ag Nanogratings	Photochemical method	5.5x (Green) and 4.6x (Red)	980 nm	^46^
Au Nanohole Arrays	E-beam lithography	35x (Green) and 34x (Red)	980 nm	^47^
Au Nanorods@SiO_2_	Seed-mediated growth modified Stöber method	3x (Green) and 6.7x (Red)	980 nm	^48^
Al Nanocylinder arrays	Nanoimprint lithography-reactive ion etching	9x (Red)	980 nm	^49^
Au Dendrites/Macro-PSi	Electrochemical	26x (Green) and 35x (Red)	800 nm	This work

## Conclusion

This work presents the first study of plasmon-enhanced upconversion photoluminescence in the $\alpha$-NaGdF_4_:Yb^3+^,Er^3+^ UC NPs coupled with gold dendritic plasmonic structures. Three types of AuDNs with different morphologies were prepared. UC NPs were deposited onto AuDNs by a simple drop-casting method. The type AuDNs-02 demonstrated outstanding performance, achieving broadband UCPL enhancement (780–990 nm excitation) and a 35-fold increased peak integrated intensity (red emission) and an 26-fold increase (green emission) compared to the reference UC NPs on silicon with 800 nm excitation. This significant enhancement is attributed to the highly localized electromagnetic fields generated by the optimized geometry of AuDNs-02 under specific excitation conditions that facilitate the LSPR effect. Based on the data obtained, we propose the plausible mechanism of the LSPR-enhanced upconversion. It should be acknowledged that further time-resolved luminescence measurements, experiments addressing distance-dependent plasmonic coupling, and study of the UCPL effect under non-resonant excitation in yttrium-free samples are of particular interest. However, it can be stated with a certain degree of confidence that the results already achieved open up prospects of a controllable PE-UCPL through the use of tailored plasmonic nanostructures. This PE-UCPL system has great potential to advance applications in bioimaging, biosensing, solar energy conversion, solid-state lighting, and display technologies.

## Experimental

### Materials

Gadolinium acetate hydrate (Gd(CH_3_COO)_3_.xH_2_O), Erbium acetate hydrate ((CH_3_COO)_3_Er.xH_2_O), Ytterbium acetate hydrate (Yb(CH_3_COO)_3_.xH_2_O), Diethylene glycol (DEG), Sodium acetate (CH_3_COONa), Ammonium fluoride (NH_4_F), Isopropyl alcohol (IPA), Chloroform, Rhodamine 6G (R6G), Hydrofluoric acid (HF, 45 %), dimethyl sulfoxide (DMSO), and Potassium tetrachloroaurate(III) (KAuCl_4_) were purchased from Sigma-Aldrich and used without additional purification.

Wafers of single-crystal silicon lightly doped with boron (*p*^–^-Si) of 12–24 $\Omega$-cm specific resistivity and (100) crystallographic surface orientation were purchased from the “Kamerton” branch of the JSC “Integral” (Pinsk, Belarus).

The water was purified with the Milli-Q system (Millipore, Bedford, MA, USA).

### Fabrication of gold-coated macroporous silicon

Macro-PSi was fabricated by electrochemical etching (anodization) of the silicon wafers in an electrolyte composed of HF and DMSO mixed at 10:48 volume ratio. The silicon wafers were cut into 3$\times$3 cm^2^ samples and placed in a fluoroplastic cell for electrochemical processing. The diameter of the active opening of the cell was 1.9 cm, allowing the formation of a round spot of macro-PSi with an area of about 3 cm^2^ on the surface of the silicon sample. Prior the anodization, the cell with the fixed silicon sample was rinsed with 20 ml of 4.5% HF for 30–60 s. This provided removal of the 2-nm thick natural SiO_2_ film^50^, which can negatively affect uniformity of the macro-PSi layer thickness. Then the cell was filled with the electrolyte for electrochemical etching of silicon, which was performed using an AUTOLAB PGSTAT302n potentiostat/galvanostat (Utrecht, the Netherlands) at a current density of 8 mA/cm^2^ for 10 min. The silicon sample acted as the anode, and the electrode in the form of a platinum wire spiral served as the cathode immersed in the electrolyte. Once anodization completed, the electrolyte was substituted with IPA for 3 h to remove products of electrochemical reactions from the pores and then rinsed with deionized water three times. Immediately after that, the cell with the macro-PSi/Si sample was filled with an electrolyte composed of 3.2 mM KAuCl_4_, 3 M C_2_H_5_OH, and HF for 40 min, which led to electroless growth of Au dendrites (AuDNs). We varied the concentration of HF in the electrolyte from 0.5 via 0.75 to 1.0 M for fabrication of three different dendritic structures (henceforth denoted as AuDNs-01, AuDNs-02, and AuDNs-03, respectively). Subsequently, the cell was washed with C_2_H_5_OH and deionized water. The AuDNs/macro-PSi sample was then extracted from the cell, desiccated in a fume hood at ambient temperature for 30 minutes, and modified with UC NPs as outlined below.

### Synthesis of the up-conversion $\alpha$-NaGdF_4_:Yb^3+^,Er^3+^ nanoparticles

$\alpha$-NaGdF_4_:Yb^3+^,Er^3+^ NPs were synthesised using the microwave-assisted hydrothermal method outlined below.

Firstly, the acetate hydrate salts of gadolinium (0.78 mM), erbium (0.02 mM) and yttrium (0.2 mM) were dispersed in 50 mL of deionized water containing 25 mL of diethylene glycol. The mixture was then stirred for about 45 minutes to obtain the A solution.

Secondly, sodium acetate (0.8 mM) and ammonium fluoride (3.2 mM) were dispersed in 30 mL deionized water containing 15 mL of IPA via an ultrasonic vibrator for 15 min, followed by magnetic stirring for 30 min in order to obtain the precursor solution. The precursor solution was added dropwise to the A solution under continuous stirring until homogeneity was achieved. Finally, the mixture was transferred to a sealed Teflon autoclave, a vessel capable of withstanding high pressure and performing the hydrothermal reaction at 130 °C for 20 minutes under pressure up to 55 atm. Thereafter, the autoclave was cooled to room temperature in a water bath for a duration of 20 minutes. The product underwent a triple filtration and washing process with deionized water, followed by a dual ethanol wash via centrifugation at 4500 rpm for a duration of 10 minutes per cycle.

### Modification of Au dendrites on macroporous silicon with up-conversion $\alpha$-NaGdF_4_:Yb^3+^,Er^3+^ nanoparticles

The as-prepared UC NPs powder was dispersed in chloroform (0.05 wt. %) via 20-min ultrasonication in order to prevent aggregation and ensure uniform distribution on the AuNDs/macro-PSi substrates. Subsequently, 5 μL of the dispersion was drop-cast onto the prepared substrates, allowing the solution to spread over its entire surface. The sample was then left to dry naturally for solvent evaporation.

### Characterization and measurements

#### Morphological, structural and optical characterization

The structural features of the AuNDs/macro-PSi substrates as well as UC NPs were examined with a Hitachi 4800 (Hitachi, Tokyo, Japan) field-emission SEM. Elemental composition analysis was performed via EDXS using a Cambridge Instruments Stereoscan-360 SEM equipped with a Link Analytical AN10000 analyzer. To reveal position of surface plasmon resonance bands of the AuDN samples, optical diffuse reflectance spectra were acquired over the 190–1000 nm range using a Specord 250 Plus spectrophotometer (Analytik Jena) coupled with an integrating sphere. The XPS analysis was performed using a Thermo Scientific K-Alpha spectrometer (USA) equipped with a hemispherical electron energy analyzer. The samples were irradiated with monochromatic Al $\textrm{K}_{\alpha }$ X-rays (1486.6 eV) that were generated at an anode voltage of 12 kV and an emission current of 3 mA. All binding energies were referenced to the adventitious carbon C1s peak at 284.8 eV. Survey spectra (0–1100 eV) were acquired with a pass energy of 100 eV and step size of 0.5 eV. The analysed area was defined by an elliptical aperture yielding an approximate surface area of 11 mm^2^.

Raman spectroscopy was used as an additional method to assess plasmonic properties of AuDNs. This was achieved by the collection of SERS spectra of R6G molecules adsorbed on the dendritic surface from the 10^–6^ M solution upon excitation with different laser wavelengths. The SERS measurements were carried out with a Confotec NR500 3D scanning laser Raman microscope-spectrometer (SOL Instruments, Belarus) equipped with 473, 633, and 785 nm lasers. A 40$\times$ objective used to focus lasers on the sample surface had a 0.65 numerical aperture (NA). The powers of the 473, 633, and 785 nm laser beams that passed through the objective were 4.65, 4.66, and 13.67 mW, respectively.

The UCPL intensity was recorded with a Confotec CARS 3D scanning laser confocal Raman spectrometer (SOL Instruments, Belarus) which was integrated with an inverted microscope (TE2000S, NIKON) and a 100$\times$ objective (NA = 0.75). The excitation source employed was a picosecond Nd: YVO_4_ laser (Ekspla, PT257-SOPO, Lithuania), operating within the 690–990 nm wavelength range, with a pulse duration of $\sim$ 6 ps, and a pulse repetition rate of 85 MHz. The laser power was maintained at 1.6 mW for UCPL measurements. Emission signals were dispersed using an MS5004i imaging monochromator-spectrograph and were detected with a ProScan HS-101H digital slow-scan CCD camera. This detection system incorporated a grating (150 grooves/mm), a short-pass filter (FF01-775/SP-25), a 100 μm pinhole, with an accumulation time of 1 s. UCPL excitation spectra were measured at a constant laser power of 9.6 mW. Excitation wavelengths were selected using a tunable laser source, with intensity controlled via neutral density filters placed prior to the objective. Power-dependent UCPL spectra were acquired under 800 nm excitation wavelength.

#### Computer simulations

The finite-difference time-domain (FDTD) simulations were performed using the Lumerical software (Vancouver, Canada).The dielectric function of gold was adopted from Johnson and Christy^51^. The AuDN was embedded in a surrounding medium of air (refractive index n = 1.0003). A total-field scattered-field source, comprising plane waves propagating backward along the z-axis with an exact wavelength of 800 nm, was employed as the excitation source. The spatial mesh size for the FDTD calculations was set to 2 nm throughout the dendritic structure. Quantitative structural parameters of the AuDNs were extracted from top-view and cross-sectional SEM images using ImageJ software.

## Data Availability

All data generated or analysed during this study are included in this published article.
